# The Gut Microbiota of Wild Mice

**DOI:** 10.1371/journal.pone.0134643

**Published:** 2015-08-10

**Authors:** Laura Weldon, Stephen Abolins, Luca Lenzi, Christian Bourne, Eleanor M. Riley, Mark Viney

**Affiliations:** 1 School of Biological Sciences, University of Bristol, Bristol, United Kingdom; 2 Centre for Genomic Research, Institute of Integrative Biology, University of Liverpool, Liverpool, United Kingdom; 3 Department of Immunology and Infection, London School of Hygiene and Tropical Medicine, London, United Kingdom; University of Camerino, ITALY

## Abstract

The gut microbiota profoundly affects the biology of its host. The composition of the microbiota is dynamic and is affected by both host genetic and many environmental effects. The gut microbiota of laboratory mice has been studied extensively, which has uncovered many of the effects that the microbiota can have. This work has also shown that the environments of different research institutions can affect the mouse microbiota. There has been relatively limited study of the microbiota of wild mice, but this has shown that it typically differs from that of laboratory mice (and that maintaining wild caught mice in the laboratory can quite quickly alter the microbiota). There is also inter-individual variation in the microbiota of wild mice, with this principally explained by geographical location. In this study we have characterised the gut (both the caecum and rectum) microbiota of wild caught *Mus musculus domesticus* at three UK sites and have investigated how the microbiota varies depending on host location and host characteristics. We find that the microbiota of these mice are generally consistent with those described from other wild mice. The rectal and caecal microbiotas of individual mice are generally more similar to each other, than they are to the microbiota of other individuals. We found significant differences in the diversity of the microbiotas among mice from different sample sites. There were significant correlations of microbiota diversity and body weight, a measure of age, body-mass index, serum concentration of leptin, and virus, nematode and mite infection.

## Introduction

The vertebrate gut microbiota is a large and diverse assemblage of bacteria that has profound effects on host individuals [1,2,3,4,5,6]. The gut microbiota is necessary for the normal development of the gut [7,8], normal physiological functioning, including immune function [9,10], and for the processing of food [11]. The development and maturation of gut immune responses requires the gut microbiota; for example, when germ-free mice were colonised with either mouse, human or rat microbiota only those colonised by mouse microbiota developed the full complement of CD4+ and CD8+ T cells, proliferating T cells, dendritic cells and antimicrobial peptide expression [12].

Individuals begin to be colonised with microbes from birth [13] and, for humans, during the first year of life individuals’ microbiotas vary. Eventually these converge to a mixture of major bacterial taxonomic groups from about one year of age, which persists through life [4,14,15]. Generally in vertebrates, once an individual’s gut microbiota is established its composition is relatively stable. There are both genetic and environmental (*e*.*g*. bacterial exposure, diet) effects on an individual’s gut microbiota (*e*.*g*. [4,9,14,15,16,17,18,19,20,21,22]). There is now compelling evidence of relationships between the gut microbiota and host disease state, which includes obesity, autoimmune disease and pathogen invasion (*e*.*g*. [2,23,24]). Individuals’ disease state can also be changed by microbiota transplantation or reconstitution (*e*.*g*. [25,26]).

The gut is also home to other organisms, including protozoa and helminth macroparasites, which interact with the bacterial microbiota. Infection of mice with the nematode *Heligmosomoides polygyrus* affects the bacterial microbiota, particularly increasing the abundance of the Lactobacillaceae family [25]. Conversely, the intestinal nematode *Trichuris muris* requires the gut microbiota to successfully establish in the gut of its host [27].

The causes of differences in gut microbiota among different individuals, groups, populations and species has been explored. A comparison of the gut microbiota from 60 species of mammals found that although there were among-individual differences within species, these differences were less than the average among-species differences [28]. Analogously, individuals’ gut microbiotas are more self-similar over time, than they are compared to those of neighbouring individuals [20]. In mice, comparison of ten genetically distinct, inbred mouse strains found that each mouse strain had a distinct bacterial community [17]. Cohabitation of different mouse strains showed that there was some convergence of gut microbiota between the strains, but that each mouse strain retained a distinct microbiota [17].

Because of the very widespread use of laboratory mice there has been extensive study of their gut microbiota, which has contributed to understanding the myriad effects that the gut microbiota can have. This work has also shown that laboratory mice obtained from different suppliers or from different research institutions can often have distinct gut microbiotas [17,29], as can genetically identical mice in different rooms within one facility [30], all of which may significantly confound experimental results. Analogously, the zebrafish gut microbiota has also been found to differ between different research facilities [31].

While there has been very extensive investigation of the gut microbiota of laboratory mice, the gut microbiota of wild house mice, *Mus musculus domesticus*, has received far less attention. Wild mice obtained from across France and Germany were compared for their microbiota, which were sampled from both the mucosa and the lumenal contents of the caeca [32,33]. These studies found that the patterns of microbiota diversity were principally explained by the geographical location of the mice (specifically the distance between the sites where the mice were sampled), with weaker effects due to the population structure of the mice and their genetic distance. Comparison of the mucosa and luminal microbiotas showed that the mucosal bacterial community within any one mouse was, on average, more similar to its own luminal microbiota than it was to the mucosal microbiota of other mice [32]. Among these wild mice two microbiota enterotypes were distinguished, characterised by differences in the occurrence of *Bacteroides* and *Robinsoniella* bacterial taxa [33]. When wild mice were transferred to the laboratory and maintained there for a year, their microbiotas were only of a single enterotype suggesting that the laboratory environment affects the composition of the gut microbiota [33]. An analogous study with a different species of rodent, the desert woodrat (*Neotoma lepida*), investigated how their gut microbiota changed over six months of captivity [34]. This found that there was a relatively small decline in measures of the diversity of the gut microbiota over six months; specifically, there was a 68% overlap in bacterial taxa between samples from wild animals and between samples from animals that had been in captivity for six months [34].

A second study of wild mice compared the gut microbiota of wild caught *M*. *musculus* with that of wild-derived, inbred strains of *M*. *m*. *musculus* and *M*. *m*. *domesticus* [35]. This found that there were substantial differences in the gut microbiota of the wild and wild-derived, inbred strains, with approximately 16% of the bacterial taxa differing between the microbiota from the wild and inbred mice [35].

The aim of this pilot study was to characterise the microbiota of wild caught *M*. *musculus domesticus* at three sample sites in the southern UK; these mice are part of a larger study investigating their ecoimmunology [36,37]. This is therefore the first report of the gut microbiota of wild mice from the UK. We were particularly interested in investigating the relationship of the caecal and rectal microbiota of the mice, and how these microbiotas varied due to sample location and host characteristics, specifically their sex, age and size as well as physiological status (their serum concentration of haemoglobin and leptin) as well as their nematode, mite and virus infection. This is the first study of which we are aware that has considered the relationships between these characteristics of wild mice and their gut microbiota.

## Materials and Methods

### Overview

As part of an on-going study [36], we sampled wild mice from various locations in the south of England and, for a sub-set of these, we chose to characterise their microbiota.

### Mice

The mice used in this study were trapped between February and May 2013 in three locations: a Gloucestershire organic dairy farm (sample prefix WF; OS reference SO 882 004); a mixed arable and beef farm south of Bristol (sample prefix HW; OS reference ST 506 671); the London Underground system (sample prefix LU; OS reference TQ 290 812). The farm owners and London Underground gave permission for trapping of the mice. Mice were trapped with Longworth traps (Penlon Ltd., UK) baited with oats, raw carrot and hay bedding. Mice caught on either farm were transferred to a conventional animal house where they were fed on a commercially available rodent diet *ad libitum* (EURodent diet 22%; PMI Nutrition International, LLC, Brentwood, MO, USA), for two to seven days before they were killed by an overdose of sodium pentobarbitone anaesthetic. Mice caught on the London Underground were taken to the lab where they were killed directly in the same way. This study was approved by the University of Bristol’s Animal Welfare and Ethical Review Board.

The mice were weighed when they were trapped. Once killed the body length of each mouse was measured from the tip of its snout to the base of its tail. From these two measures we then calculated the weight / body length ratio, which is effectively a mouse body-mass index. Each mouse was then dissected and the fat present in the abdominal cavity was removed, weighed and stored at -20°C. The caecum and large intestine were removed and stored at -20°C. We also removed the eye lenses of each mouse and processed them as described before [38] and then determined the lens dry weights, which is a validated measure of mouse age [38]. A total of 14 mice were used and their details are shown in Table 1.

**Table 1:** A description of the 14 trapped mice and their infection status.

Trap site	ID	Trapped weight (g)	Days housed	Sex	Virus^1^	Mite number^2^	Nematode burden^3^
HW	248	11	6	F	3	101–200	34
HW	277	15	6	M	2	401–500	46
HW	280	16	6	M	4	401–500	74
HW	282	13	7	M	2	401–500	317
HW	309	18	7	F	3	401–500	5
LU	261	19	0	F	7	0	0
LU	265	19	0	M	5	0	0
LU	271	18	0	M	6	41–50	0
LU	273	18	0	F	7	0	0
WF	260	9	5	F	2	51–100	37
WF	290	21	5	F	3	51–100	44
WF	291	7	5	M	0	51–100	156
WF	292	8	5	F	0	101–200	124
WF	303	10	2	F	1	401–500	213

^1^ Number of infections from a possible 6 virus and one bacterial infection;

^2^ *Myocoptes musculinus*;

^3^ *Syphacia* sp.

Blood samples were taken from each killed mouse and the haemoglobin concentration measured using a HemoCue Hb 201 analyser (HemoCue AB, Ängelholm, Sweden). We also measured the serum concentration of leptin of each mouse using a commercially available kit (Insight Biotechnology Ltd., UK), and assayed each mouse for evidence of microbial infection using two immunocomb kits (Biogal Galed Labs, Israel), which detect antibodies to the Corona, Mouse Hepatitis, Sendai, Minute, Noro and Parvo viruses and to *Mycoplasma pulmonis*.

### Parasites

We determined the ectoparasite fauna of the caught mice. Ectoparasites were counted by a visual inspection of killed mice and, for large parasites such as fleas and ticks, the trap contents were also inspected. For each mouse, the total skin surface and fur were examined under a dissecting microscope. Ectoparasite samples were removed for identification and the number of mites observed was classified into the categories: 0–10 recorded as the actual number, 11–20, 21–30, 31–40, 41–50, 51–100, 101–200, 201–300, 301–400, 401–500, 501–1000, 1001–1500, 1501–2000. Mites were identified as *Myocoptes musculinus* by morphological examination [39].

We also determined the intestinal nematode fauna of the caught mice. To do this, the stored intestines (see above) were defrosted, slit longitudinally and the gut examined using a dissecting microscope and worms counted as described previously [40]. Detailed morphological examination of a representative sample of the worms identified them as nematode pinworms *Syphacia* sp.

### DNA preparation and amplification

For each mouse (Table 1) the caecum and large intestine were defrosted and 250mg of caecal and rectal contents from each were removed to separate tubes from which bacterial DNA was extracted using the QIAmp DNA stool mini kit (Qiagen), and the resulting DNA was quantified using a Qubit fluorometer (Thermo Fisher Scientific).

From the 14 mice a total of 39 samples were PCR amplified as: (i) one caecal and one rectal sample from each of eleven mice–a total of twenty two samples, and (ii) three technical replicates (*i*.*e*. PCR amplification of DNA) of a caecal and rectal sample from each of three mice (HW248, HW277, HW280, Table 1; one caecal replicate of HW248 was excluded due to a technical error)–a total of seventeen samples. Together, this generated 39 samples.

DNA was amplified with universal primers for the V4 and V5 regions of the 16S rRNA gene U515F (5′-GTGYCAGCMGCCGCGGTA) and U927R (5′-CCCGYCAATTCMTTTRAGT) as described by [41], but the forward primer was modified at the 5’ end by the addition of a ten base nucleotide multiplex identifier (MID) (of which there were a total of 21 unique MIDs), followed by a four base key and finally an adapter that was required for sequencing.

### DNA sequencing and bioinformatics analyses

High throughput DNA sequencing and subsequent bioinformatic analyses were carried out by the Centre for Genomic Research, University of Liverpool. Two sequencing pools were generated, consisting of 21 and 18 MID-indexed amplicons and pyrosequenced on a 454 GS FLX+ platform using xlr70 (Titanium) chemistry. All the described analysis steps were performed using scripts from the Qiime v1.8.0 package [42]. Briefly, reads from the two, pooled datasets were filtered to remove short, long and error-prone reads and separated into their respective samples based on MIDs. This was done using the script split_libraries.py with default parameters, except for the length thresholds which were set to remove reads either shorter than 350bp or longer than 450bp. De-multiplexed reads were de-noised using Denoiser [43] to reduce sequencing-introduced errors, which therefore reduces any potential overestimation of diversity. The Qiime scripts denoise_wrapper.py and inflate_denoise_output.py were used to do this. These error-free sequences were pooled into a single file containing 1,245,093 sequences from the 39 samples. The Greengenes rRNA database version 12.10 [44], clustered at 97% of identity, was used in the chimera detection step and the taxonomic assignment step, obtained from the Qiime website (qiime.org/home_static/dataFiles.html).

In order to define operational taxonomic units (OTUs) sequences were clustered to 97% similarity, which included additional steps to account for base errors, error correction, and for chimeras. All these steps were performed using the script pick_otus.py, which utilises USEARCH5 [45] to perform error correction, clustering and chimera filtering steps. Chimeric OTUs were identified *de novo* among the read set and using a database of known 16S sequences as reference; only OTUs passing both filters were retained.

Clustering at thresholds greater than 97% similarity for pyro-sequencing reads tends to inflate diversity estimates [46]. Because errors are expected to be rare, a final filter was introduced to remove clusters containing fewer than four sequences, since such clusters are more likely to be a result of errors, compared with more highly populated clusters. This resulted in 1,004 sequences passing all filtering steps, each of them identifying a putative OTU within the dataset. The abundance of each of the identified OTUs was computed using the error-free read set and a similarity threshold of at least 97%. A representative sequence for each OTU was defined as the one that was most abundant among the sequences in that cluster, using the pick_rep_set.py script. The taxonomy at species level for each representative sequence was identified using the RDP classifier (version 2.2, [47]). This process was performed by the Qiime script assign_taxonomy.py.

The file containing the OTU abundance information (in biom format, http://biom-format.org/), the metadata of the experimental design, the final filtered OTU representative sequence set and its phylogenetic tree, all obtained from the Qiime pipeline, were imported into the R environment on a Debian / Linux computer and analysed using the Phyloseq package [48].

To calculate the alpha diversity of the samples we used the Chao 1 richness measure [49].

The alpha diversity was calculated separately for caecal and rectal samples. Because both of these samples were taken from each mouse they could not be considered as independent replicates during the various analyses, all of which were done using SPSS v.21, IBM Corp., New York, USA.

The Chao1 alpha diversity values for each sample were then analysed with univariate general linear models (GLM) to investigate any effects of mouse trapping location (*i*.*e*. sites LU, WF and HF) and sex in a full factorial analysis. One model was run with caecal alpha diversity as the dependent variable and another model was run with rectal alpha diversity as the dependent variable. For the mice that were used as technical replicates, and consequently had three replicate values, the mean of these was used for analysis. Tukey’s post-hoc test was used to determine significant differences between the three trapping locations. All data were normally distributed. Sequencing pool could not be included in the univariate GLM because it was confounded with trapping location; sequencing pool 1 contained HW only, while sequencing pool 2 contained LU and WF. Therefore two separate ANOVAs were conducted for caecal alpha diversity and rectal alpha diversity to test for any effect of sequencing pool.

We searched for correlations (Pearson with two-tailed significance tests) among the Chao 1 values and measures of the mice, specifically their weight, body length, weight / body length ratio, weight of abdominal fat, eye lens weight, haemoglobin concentration, number of viral / bacterial infections, and intensity of infection with nematodes and mites. Nematode intensity data were log_10_ transformed prior to analysis.

For samples with low Chao 1 values (<200) we used the Qiime script 'multiple_rarefaction' to randomly sub-sample reads for each sample (up to a maximum of the actual number of reads obtained for that sample) and to recalculate the Chao 1 value. These data were then inspected to determine if a sample’s Chao 1 values had saturated for the given number of sequence reads.

We calculated the taxonomic dissimilarity among the samples using the Bray-Curtis metric [50] which was calculated in R, and these values were then used to calculate a nearest-neighbour joining tree in MEGA v.6.06 [51].

## Results and Discussion

We characterised the caecal and rectal microbiota of wild caught *Mus musculus domesticus* sampled from three UK sites. In total we identified 1,004 bacterial OTUs across all samples, with the average of 20,577 reads *per* sample that were associated with any OTU (SD 11,063, range 3,846–50,279).

**Fig 1:**
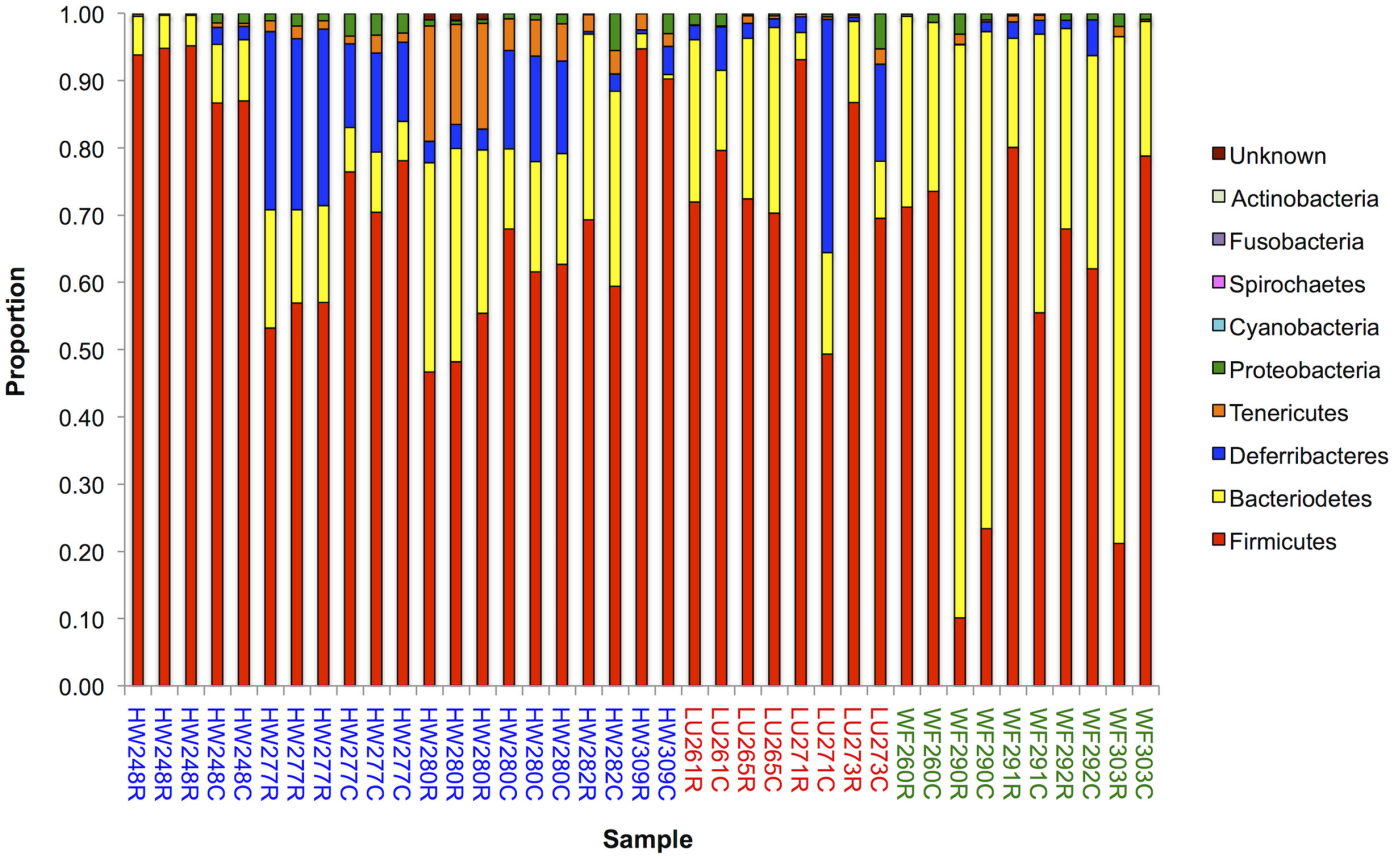
Bacterial Phyla. A histogram showing the relative proportion of different bacterial phyla present in 39 samples from 14 mice, where the two letter prefix denotes the location from where the mouse was captured and the three digit number is the unique mouse identifier; C and R are caecal and rectal samples, respectively, all as Table 1.

Among the 39 samples from 14 mice, the most abundant bacterial phyla were Firmicutes and Bacteroidetes (Fig 1), with these representing a mean of 68% (SD 20%) and 22% (SD 19%), respectively, of the taxa present. Three bacterial phyla comprised the majority of the remaining taxa present in our samples; specifically, Deferribacteres, Tenericutes and Proteobacteria (mean and (SD) 7 (9); 2 (4); 1(1)%, respectively).

These results are consistent with other studies of mice where *c*.90% of the mouse distal microbiota belonged to the Firmicutes and Bacteriodetes phyla [3,32,35]. Bacteria from the Firmicutes and Bacteroidetes phyla usually dominate in a healthy vertebrate host and are responsible for a variety of roles, including the generation of metabolites, immune system maturation, angiogenesis and fat storage [7,52,53,54,55]. This is the first report of the gut microbiota of mice from the UK.

In our samples the relative proportion of Firmicutes and Bacteriodetes varied among the samples (Firmicutes range 10–95%; Fig 1), but with Firmicutes dominating in all but one case (see below). Overall our results are consistent with other studies that found that Firmicutes were the dominant taxa in wild mice, but that Bacteroidetes was dominant in inbred laboratory strains [35]. The relative abundance of these two phyla has been found to alter depending on food source, with fasting increasing the relative proportion of Bacteroidetes, and a higher relative proportion of Firmicutes being associated with obese individuals [55]. Among our mice, different individuals will likely have different access to food (especially when in high density populations where there is likely to be density-dependent competition for resources) that may underlie the variation in the abundance of Firmicutes bacteria seen among our mice.

**Fig 2:**
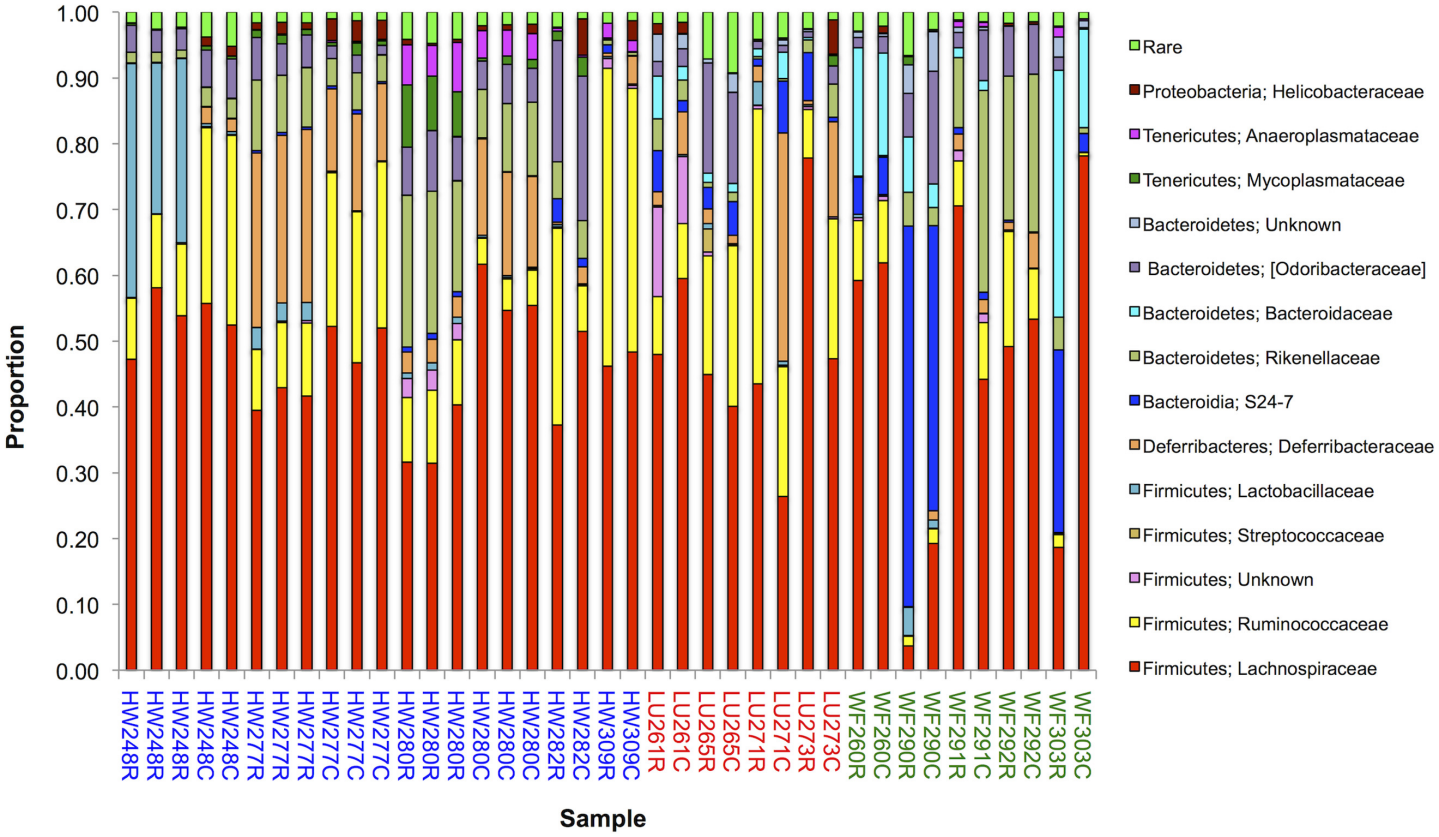
Bacterial Families. A histogram showing the relative proportion of different bacterial families present in 39 samples from 14 mice, where the two letter prefix denotes the location from where the mouse was captured and the three digit number is the unique mouse identifier; C and R are caecal and rectal samples, respectively, all as Table 1. For a bacterial family to be shown its abundance was ≥3% in at least one sample; bacterial families whose abundance was below this criterion are grouped in the category ‘rare’.

At the bacterial family level, Lachnospiraceae and Ruminococcaceae (both Firmicutes) were the most common (mean abundance of 47% (SD 15%) and 15% (SD 11%), respectively) (Fig 2). Taxa of these families are associated with the maintenance of gut health and, while they contain functionally diverse taxa, they share a common role as active degraders of plant-derived material in the gut [56]. Our identification of OTUs by sequence homology to family level was very high, consistent with previous work showing that unknown or poorly described microbes in wild house mice are comparatively rare [35].

For three mice (HW248, HW277, HW280, Table 1) there were up to three technical replicates of each of their caecal and rectal samples. There was a close concordance of the bacterial abundance at the phylum and family level among the technical replicates of each mouse for each caecal or rectal sample (Figs 1 and 2). Among these three mice, the bacterial abundance differed markedly between the rectal and caecal samples, and this pattern is generally seen among the other 11 mice too (Figs 1 and 2). This is most notable at the bacterial family level analysis, especially for taxa other than Lachnospiraceae (Fig 2). The extent of the difference between rectal and caecal samples varies, such that in some mice they were very different (*e*.*g*. WF303 and LU271), while in others they were more similar (*e*.*g*. HW309, WF260) (Figs 1 and 2).

**Fig 3:**
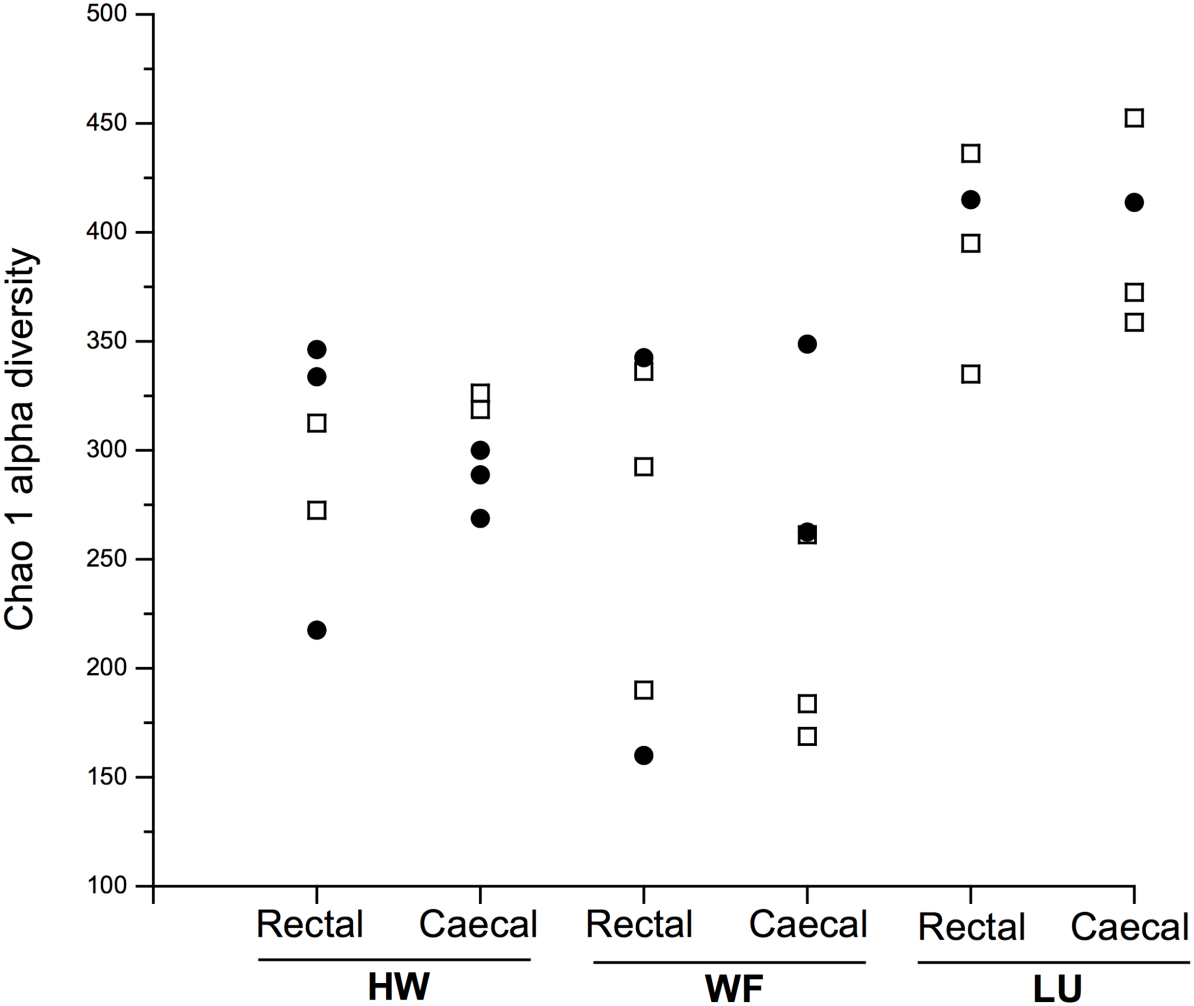
Chao 1 alpha diversity. The Chao 1 alpha diversity values for mice at the three trapping locations (HW, WF, LU) for rectal and caecal samples where each data point represents one mouse; ⬜ female, ● male.

We investigated whether mouse trapping location, sex, or sequencing pool affected the Chao 1 alpha diversity values of the caecal and rectal samples. There was a significant effect of trapping location on the caecal alpha diversity values (*F*_2, 12_ = 11.49, *p* = 0.004; Fig 3, Table 2). Samples from mice from the LU site had significantly greater alpha diversity than samples from HW (*p* = 0.020) and WF (*p* = 0.002), but there was no significant difference between samples from HW and WF (*p* = 0.158). However, there was no significant effect of trapping location on the Chao 1 alpha diversity of the rectal samples (*F*_2, 11_ = 2.85, *p* = 0.125; Fig 3, Table 2). Previous studies have found geographical effects on measures of wild mouse microbiota, [32] and our results are therefore broadly consistent with these. The cause of the differences among sample sites is not known, but could be due to any systematic differences among the mice from different sample sites (*e*.*g*. their genetics, infection status, habitat, body condition *etc*.); though equally there may be a reverse causality, such that different infection status, habitat and body condition might be driving the differences in the diversity of the mouse microbiotas. Further study of more mice would be needed to understand these likely complex relationships. Studies of human gut microbiota have shown that these are generally stable, but that behavioural and lifestyle changes can bring about short-term perturbations to the microbiota [57].

**Table 2:** Mean Chao 1 alpha diversity (± SD) values for each trapping location. Letters after the values denote groups that differ significantly at *p* < 0.01 for caecal alpha diversity; there were no significant differences for the rectal alpha diversity.

Trap site	Chao 1 diversity (SD)	
	Caecal	Rectal
LU	399.5 (42.3)_a_	395.0 (43.4)
HW	300.5 (23.0)_b_	296.9 (52.5)
WF	245.1 (72.4)_b_	290.8 (70.5)

Four samples (WF291R, WF292C, WF303C, WF303R) had Chao 1 values that were less than 200, and we tested (using the Qiime multiple rarefaction procedure) whether this was because of insufficient sample reads to have saturated the Chao 1 values. This showed that for sample WF291R, with 3,486 reads, its alpha diversity was potentially underestimated; for all other samples there was a sufficient number of sequence reads to measure the alpha diversity. WF291R was thus excluded from all analyses.

There were no significant effects of mouse sex on either caecal or rectal alpha diversity. Sequencing was performed in two pools, one containing all of the HW samples and the second containing the others (Table 1). There was no significant effect of sequencing pool on caecal alpha diversity (*F*_1, 12_ = 0.084, *p* = 0.777) or rectal alpha diversity (*F*_1, 11_ = 1.34, *p* = 0.271), although it is not possible to fully disentangle potential effects of HW from potential effects of sequencing pool.

For the sampled mice we also had a range of other measures of these animals, including body weight, body length, (and thus weight / body length ratio), dry eye lens weight (a measure of age [38]), the weight of abdominal fat, the serum concentration of leptin, measures of their infection status with parasitic nematodes and mites, and antibody-based evidence of microbial infection. We sought correlations of these measures with the Chao 1 measure of alpha diversity (Table 3). This showed significant correlations between the alpha diversity of the caecal and the rectal samples with virus infection, with the intensity of nematode infection and the intensity of mite infection. We note that the number of virus infections was positively correlated with caecal and rectal alpha diversity, but that the intensity of macroparasite (nematode and mite) infections was negatively correlated with microbiota diversity. Further, the worm, mite and virus infection was different in the London Underground mice compared with the other sites (Table 1); specifically, mice from the London Underground had evidence of more viral infections compared with the other mice, but had no nematode infection (which was almost ubiquitous in mice from other sites) and almost no mites. There has been relatively limited laboratory-based study of how infection affects mouse microbiota, but effects have been found (*e*.*g*. [25]). Our results are therefore of significance because they suggest the likely important effects of wild animals' infections on their microbiota. These infection differences may, in part, explain the site differences in alpha diversity (Fig 3).

**Table 3:** Correlation coefficients (and *p* value) between Chao 1 alpha diversity values for the caecum and rectum and a range of measures of the mice and their infection status. Sample WF291R is excluded (see above). Significant effects are shown as ** *p* ≤ 0.01, * *p* ≤ 0.05.

	Alpha diversity	
	Caecum (n = 14)	Rectum (n = 13)
**Weight (g)**	0.54 (0.042)*	0.59 (0.033)*
**Body length (cm)**	0.51 (0.061)	0.47 (0.102)
**Weight / Body length**	0.52 (0.053)*	0.59 (0.034)*
**Abdominal fat (g)**	-0.12 (0.690)	-0.061 (0.842)
**Eye lens weight (g)**	0.61 (0.020)*	0.63 (0.019)*
**Leptin (ng/mL)**	-0.83 (0.005)**	-0.59 (0.093)
**Haemoglobin (g/L)**	0.22 (0.436)	-0.11 (0.713)
**Virus** ^1^	0.73 (0.007)**	0.71 (0.009)**
**Mites** ^2^	-0.54 (0.048)*	-0.64 (0.018)*
**Nematode** ^3^	-0.66 (0.010)**	-0.70 (0.07)**

Number of

^1^microbial infections,

^2^ *Myocoptes musculinus*, and

^3^ *Syphacia* sp.

The body weight and dry eye lens weight of the mice were also both significantly positively correlated with the alpha diversity of the caecal and rectal samples (Table 3). There has been considerable interest in the relationship between gut microbiota and obesity in humans [1,2,21,55], and it is of note that we also find that the caecal and rectal microbiota alpha diversity is significantly positively correlated with the ratio of body weight to body length (effectively a body-mass index), and that the caecal microbiota alpha diversity is significantly negatively correlated with the serum concentration of leptin. These data are the first, of which we are aware, to investigate how these characteristics of wild mice are related to measures of their microbiota. Laboratory-based studies have sought to understand what affects the gut microbiota, and our results are therefore important because they suggest that analogous processes are occurring beyond the laboratory in wild animals. We have a relatively small sample size of mice and this precludes us from being able to further un-pick the relationships among these multiple measures of the biology, condition and infection status of these wild mice, but this could be done by investigating a larger sample of mice.

**Fig 4:**
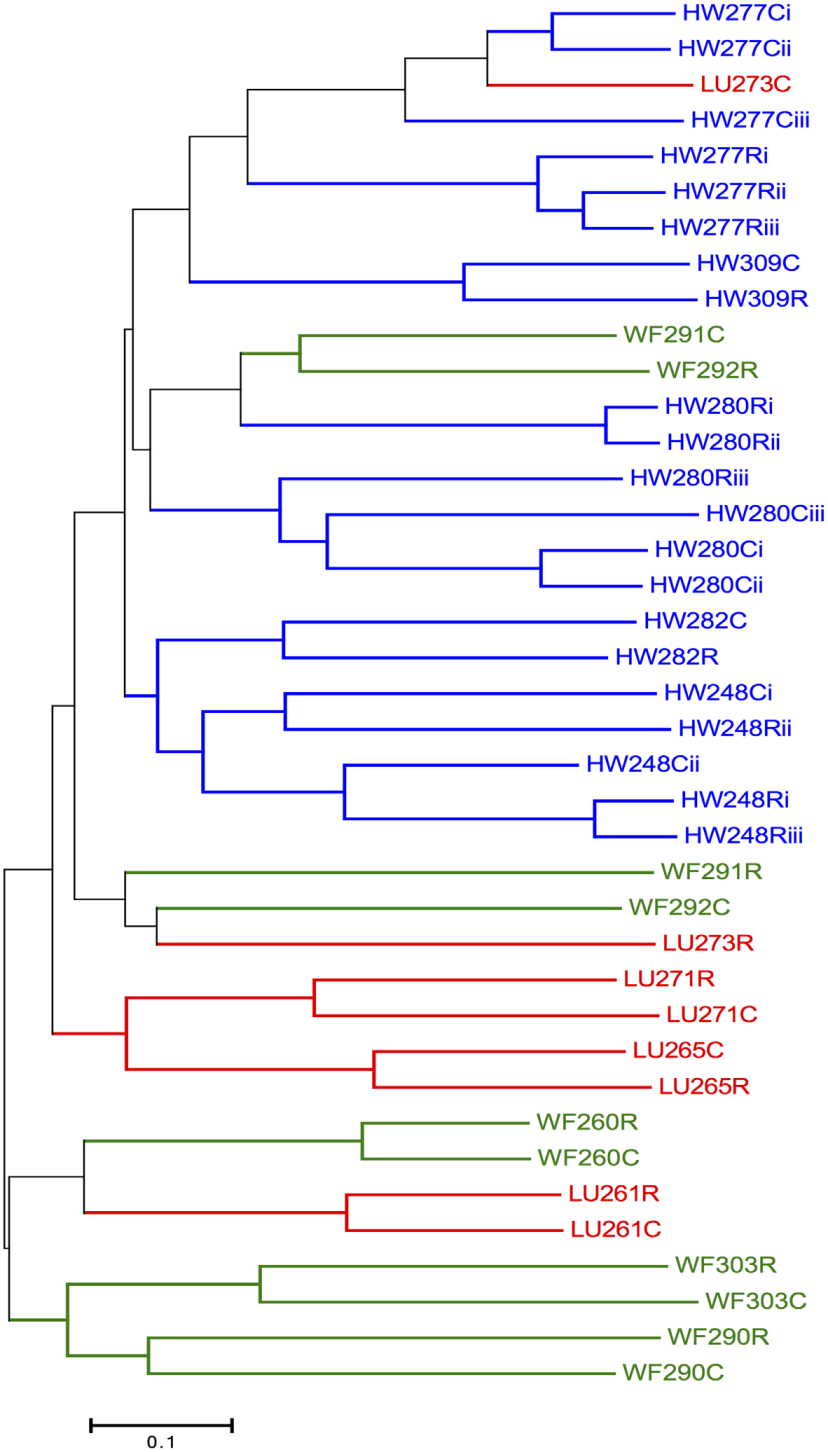
Bray-Curtis dissimilarity. A dendrogram showing the dissimilarity among the 39 samples from 14 mice, based on the Bray-Curtis measure of dissimilarity (range 0–1), with samples colour-coded as blue HW, green WF, and red for LU; C and R are caecal and rectal samples, respectively, all as Table 1. Subscripts i, ii, and iii refer to technical replicates for the respective samples.

The Bray-Curtis distance data was used to measure the compositional dissimilarity between all the samples from the mice, including the technical replicates from individual mice (Fig 4). This shows that generally the caecal and rectal samples for individual mice are usually closely clustered. In general therefore, the caecal and rectal samples of a mouse are more similar to each other than they are to samples from different mice. While this was the predominant pattern, there were some exceptions, such as mouse LU273 where the caecal and rectal microbiotas differ considerably by this measure.

Two mice (WF290 and WF303) had microbiotas that were notably distinct from the other mice (Figs 1 and 2). Both of these mice were in poor health at time of capture and had the high nematode infection intensities (Table 1). The spleen of WF303 was degenerate, appearing small and blackened. This evidence of ill health of these individuals may be related to their notably distinct microbiotas, also consistent with studies showing how an otherwise stable microbiota can be perturbed [56].

The mice we sampled were maintained in the laboratory for between two and seven days (Table 1), but the effect of this, if any, on their microbiota is unclear. Other studies have found that longer periods of laboratory maintenance can affect the gut microbiota, particularly when the microbiotas were classified by enterotype [33]. Also, over much shorter periods, specifically for 1–12 weeks of laboratory housing, these same changes (converging to one enterotype) could be observed too, although changes in abundance of individual bacterial genera were generally less marked [33]. The short-term housing of our mice may therefore have had an effect on the gut microbiota that we have described here.

This work was a pilot study of the gut microbiota of wild mice from the south of England. This is the first such study of wild mice from the UK and the first study that has investigated how characteristics of the mice (their geographical site, body size, age, physiological state and infection status) is related to their microbiota. We find notable differences in the microbiota between mice from different sample sites, and significant relationships among the microbiota and mouse weight, weight / length ratio, a measure of age, the serum concentration of leptin, as well as nematode, mite and virus infection. A study of the gut microbiota of the other approximate 500 mice that have been sampled could be undertaken to further characterise the diversity of the microbiota and dissect apart the factors that affect the mouse microbiota and, equally, how the microbiota affects aspects of the wild biology of these mice. This work has found that the microbiota of these mice is broadly similar to that of other wild mice, but that there is a diversity in the microbiota that is likely to be of biological significance.
